# Progression of Parkinson's disease is associated with gut dysbiosis: Two-year follow-up study

**DOI:** 10.1371/journal.pone.0187307

**Published:** 2017-11-01

**Authors:** Tomomi Minato, Tetsuya Maeda, Yoshiro Fujisawa, Hirokazu Tsuji, Koji Nomoto, Kinji Ohno, Masaaki Hirayama

**Affiliations:** 1 Department of Pathophysiological Laboratory Sciences, Nagoya University Graduate School of Medicine, Nagoya, Japan; 2 Division of Neurology and Gerontology, Department of Internal Medicine, School of medicine, Iwate Medical University, Morioka, Japan; 3 Yakult Central Institute, Tokyo, Japan; 4 Division of Neurogenetics, Center for Neurological Diseases and Cancer, Nagoya University Graduate School of Medicine, Nagoya, Japan; Okayama University Graduate School of Medicine, Dentistry and Pharmaceutical Sciences, JAPAN

## Abstract

**Background:**

We previously reported gut dysbiosis in patients with Parkinson’s disease (PD).

**Objective:**

The aim of this study is to examine whether gut dysbiosis correlates with the progression of PD.

**Methods:**

We examined changes in gut microbiota and demographic features in 2 years in 36 PD patients.

**Results:**

A change of total UPDRS scores in 2 years was predicted by the counts of *Bifidobacterium* and *Atopobium* cluster at year 0 with a correlation coefficient of 0.52. Correlation analysis additionally revealed that low counts of *Bifidobacterium* and *Bacteroides fragilis* at year 0 were associated with worsening of UPDRS I scores in 2 years. In addition, low counts of *Bifidobacterium* at year 0 were associated with worsening of hallucinations/delusions in 2 years. Similarly, low counts of *B*. *fragilis* at year 0 were associated with worsening of motivation/initiative in 2 years. The patients were evenly divided into the deteriorated and stable groups based on the degree of worsening of total UPDRS scores. The deteriorated group had lower counts of *Bifidobacterium*, *B*. *fragilis*, and *Clostridium leptium* than the stable group at year 0 but not at year 2, suggesting that the deteriorated group may demonstrate accelerated lowering of these bacteria at year 0.

**Conclusions:**

The total counts of intestinal bacterial decrease in the course of PD progression. Temporal profiles of lowering of bacterial counts are likely to be different from bacteria to bacteria, and also between the deteriorating and stable groups, which may be able to be exploited to differentiate patients with rapidly and slowly progressive PD pathology.

## Introduction

Parkinson’s disease (PD) is one of the common neurodegenerative disorders clinically characterized by motor and non-motor symptoms in aged individuals. PD is predicted to affect more than 10 million people worldwide by the year 2030 [1]. Pathohistological hallmark of PD is the presence of abnormally aggregated α-synuclein (Lewy bodies) in the central nervous system (CNS). Lewy body pathology in PD is found in various regions in the CNS, particularly in the substantia nigra. Lewy body pathology is also observed in the peripheral autonomic and enteric nervous system (ENS) in the gastrointestinal tract, the olfactory system, and the cardiac sympathetic system, which suggests that α-synuclein pathology in PD may start in these tissues [2]. PD patients frequently exhibit non-motor symptoms, including signs and symptoms of gastrointestinal dysmotility (e.g. delayed gastric emptying [3] and constipation [4]). Similarly, in PD, accumulation of α-synuclein in the ENS could commence 20 years before the onset of degenerative changes in the CNS and the associated motor symptoms [5]. In accordance with these observations, the smell test [6] and cardiac meta-iodobenzylguanidine scintigraphy [7] are sensitive methods to diagnose early PD. In addition, constipation is the most common premotor symptom in PD, and constipation antedates the development of parkinsonian symptoms by 10 or more years [8]. These findings suggest that Lewy body pathology in the ENS might cause gastrointestinal symptoms in PD. In PD, intestinal permeability is increased, and is correlated with increased intestinal staining for *Escherichia coli*, α-synuclein, and nitrotyrosine, which is a marker for protein oxidation [9]. Similarly, intraperitoneal administration of lipopolysaccharide (LPS) induces hyperpermeability of the large intestine, and abnormal aggregation of phosphorylated α-synuclein in the large intestine [10]. Additionally, transplantation of gut microbiota of 6 PD patients to transgenic mice over-expressing α-synuclein causes worsening of motor deficits [11]. Oxidative stress produced by macrophages in the luminal wall due to a hyperpermeabilized intestinal wall may account for the accumulation of α-synuclein in the intestinal mucosa. As the intestinal microbiota is likely to have a substantial effect on the hyperpermeability-induced oxidative stress, the intestinal microbiota may be causally associated with α-synuclein pathology in the ENS in PD.

Intestinal dysbiosis in PD has been reported in nine articles from six countries: three from Germany [12–14]; two from the USA [15, 16]; and one from Finland [17], Japan [18], Russia [19], and China [20]. The first report was from Finland, in which *Enterobacteriaceae* were more abundant in PD with a PIGD (postural instability and gait difficulty) phenotype than in tremor-dominant PD patients [17]. The second report was from the USA, in which *Lachnospiraceae* was negatively correlated with disease duration [15]. We reported in Japan that the count of *Lactobacillus gasseri* subgroup was high in advanced PD patients, and the count of *Clostridium coccoides* group was high in early PD patients [18]. We also reported that bacteria associated with constipation are different from those associated with disease duration. Gut dysbiosis shared by most but not all reports is that *Prevotella* and butyrate-producing bacteria are decreased in PD. These reports support the notion that gut dysbiosis is associated with the development and progression of PD. In order to identify gut microbiota that are associated with progression of PD, we analyzed changes in gut microbiota in PD in 2 years. We also correlated gut microbiota at year 0 with the progression of PD in 2 years.

## Patients and methods

### Patients

All studies were approved by the ethical review committee of the Nagoya University Graduate School of Medicine (approval #2013–0047). We started our study in 2013, and followed up the patients for 2 years. All studies were performed after appropriate signed informed consents were given by all subjects. Thirty-six PD patients at the Nagoya University Hospital and at the Aichi Chapter of the Japan Parkinson’s Disease Association were able to be followed up for 2 years (22.7 ± 1.7 months, mean and SD). Mean age of the 36 PD patients was 68.6 ± 7.1 years old (mean and SD; range 46–81 years). The Hoehn and Yahir stage was 2.6 ± 0.8 (mean and SD). At year 0, the stool samples were analyzed in 36 patients. At year 2, the stool samples were analyzed in 28 patients. In one patient, the amount of the stool sample at year 2 was too low to be analyzed. In 7 other patients, patients’ family could not access the patients’ stools, because they were admitted to nursing homes.

### Clinical assessments

At years 0 and 2, the severities of PD were assessed using the Hoehn and Yahr scale (HY) [21], the Unified Parkinson’s Disease Rating Scale (UPDRS) parts I-IV [22], the Mini Mental Sate Examination (MMSE) [23], the Japanese version of the Montreal Cognitive Assessment (MoCA-J) [24], the Frontal Assessment Battery at bedside (FAB) [25], and the Odor Stick Identification Test for the Japanese (OSIT-J) [26]. As all patients were outpatients, UPDRS III and HY were evaluated 2–3 hrs after medication and assessed in the ON phase. L-dopa-equivalent dose (LED) was used to evaluate the amount of anti-parkinsonian agent. As fermented milk produced by *Lactobacillus* is widely consumed in Japan, we asked the frequency of its consumption by a questionnaire. The other alimentary habits were not questioned. Constipation was assessed by stool frequency in a week.

### Measurement of lipopolysaccharide-binding protein (LBP)

LBP levels were measured in the serum using the enzyme-linked immunosorbent assay (ELISA) kit from Hycult Biotech (HK315-01). Serum was stored at -80°C until assays were performed.

### Bacterial counts by rRNA-targeted quantitative reverse-transcription PCR (qRT-PCR)

Fecal sample was placed directly into a tube (~1.0 g/tube) containing 2 mL RNAlater (Ambion) to stabilize RNA at the participant’s home. The samples were delivered to Nagoya University, and were blindly transported to the Yakult Central Institute at 4°C. The composition of gut microbiota was analyzed using the Yakult intestinal Flora-SCAN (YIF-SCAN), which exploited qRT-PCR of bacterial 16S or 23S rRNA using SYBR Green I [27–31]. The 19 bacterial groups/genera/species were comprised of (i) 6 anaerobic species that predominate in our intestine (*Clostridium coccoides* group, *Clostridium leptum* subgroup, *Bacteroides fragilis* group, *Bifidobacterium*, *Atopobium* cluster, and *Prevotella*); (ii) 5 potential pathogens (*Clostridium perfringens*, Enterobacteriaceae, *Enterococcus*, *Staphylococcus*, and *Pseudomonas*), and (iii) 8 *Lactobacilli* (*L*. *gasseri* subgroup, *L*. *brevis*, *L*. *casei* subgroup, *L*. *fermentum*, *L*. *plantarum* subgroup, *L*. *reuteri* subgroup, *L*. *ruminis* subgroup, and *L*. *sakei* subgroup) [30, 31]. These bacteria were selected based on high prevalence in the human intestines [category (i)]; frequently observed pathogens [category (ii)]; and preference of the Yakult company that merchandises *Lactocbacillus*-containing yoghurt [category (iii)]. The 6 most prevalent bacteria in category (i) constituted 71.3 ± 9.4% (mean and SD) of total intestinal bacteria [31]. The primer sequences used for these analyses are listed in S1 Table.

We previously confirmed that the copy number of 16S or 23S rRNA by qRT-PCR was correlated to the actual count of individually culture bacteria [30, 31]. The advantage of YIF-SCAN is quantification of the exact cell numbers of 19 bacterial groups/genera/species including rare bacteria. Another advantage of rRNA analysis over rDNA analysis is that only live bacteria can be counted. On the other hand, the disadvantage of rRNA is that the numbers of rRNA is possibly affected by bacterial metabolisms, and that only a limited number of intestinal bacteria can be analyzed.

### Statistical analysis

All analyses were performed with the JMP Pro 13 statistical package (SAS Institute). The normality of data distribution was assessed by the scatter diagram and the Shapiro–Wilks test. Wilcoxon signed-rank test was used to compare datasets at years 0 and 2. Mann-Whitney U test was used to compare datasets between the stable and deteriorated groups. Gender, habitual consumption of fermented milk, detection rates of gut microbiota were analyzed using the Fisher’s exact test. A stepwise analysis was used to develop a linear regression model for predicting a change of UPDRS score in 2 years using the counts of 10 bacterial groups/genera/species, assuming that there were no confounders among the 10 bacterial groups/genera/species. When the count of *L*. *brevis* was below the detected threshold of our qRT-PCR analysis, the sample was omitted from the calculation of a Pearson’s correlation coefficient between the serum LBP levels and the counts of *L*. *brevis*. Results are considered statistically significant when *p*-value was less than 0.05. A dataset with the correlation coefficient greater than 0.30 was taken into account. For multiple comparisons of bacterial counts between the deteriorated and stable groups, the false discovery rate (FDR) was calculated using the Benjamini and Hochberg method.

## Results

### Clinical and laboratory features: Comparison between the deteriorated and stable groups

The 36 patients were evenly divided into 2 groups based on a change in the total UPDRS scores in 2 years. Each group was comprised of 18 patients. Clinical features and serum LBP levels were compared between the deteriorated and stable groups (Table 1). Total UPDRS scores were higher in the deteriorated group than the stable group even at year 0 (*p* < 0.05). The difference in UPDRS scores between the 2 groups became more prominent at year 2 (*p* < 0.01). Gender, age, duration, and HY were not different between the 2 groups at year 0. The ratios of patients with habitual consumption of fermented milk were not different between the 2 groups at years 0 and 2. MMSE, MOCA-J, and FAB were not different between the 2 groups at years 0 and 2. Similarly, serum LBP levels were not different between the 2 groups at years 0 and 2. LED tended to increase in two years in both groups but without statistical significance.

**Table 1:** Comparison of demographic features between the deteriorated and stable groups.

	Deteriorated group at year 0 (A) (*n* = 18)	Stable group at year 0 (B) (*n* = 18)	Deteriorated group at year 2 (C) (*n* = 11)	Stable group at year 2 (D) (*n* = 17)	A-B *p*-value	C-D *p*-value	A-C *p*-value	B-D *p*-value
Age (years)	70.2 ± 5.6	67.0 ± 8.2			n.s.	-	-	-
Gender (%)					n.s.	-	-	-
F	44.4	77.8						
M	55.6	22.2						
Disease duration (years)	9.2 ± 4.6	9.8 ± 6.1			n.s.	-	-	-
Habitual consumption of fermented milk (ratio)	15/18	13/18	11/11	15/17	n.s.	n.s.	n.s.	n.s.
BMI	20.5 ± 2.7	19.6 ± 2.4	21.7 ± 2.5	19.4 ± 2.6	n.s.	n.s.	n.s.	n.s.
UPDRS I	3.2 ± 2.4	2.4 ± 1.9	5.1 ± 3.8	1.9 ± 1.9	n.s.	n.s.	< 0.005	n.s.
UPDRS II	12.3 ± 7,2	9.4 ± 5.7	21.8 ± 8.5	11.9 ± 6.4	n.s.	< 0.01	< 0.0001	n.s.
UPDRS III	28.9 ± 13.5	21.6 ± 8.1	36.5 ± 15.9	20.2 ± 8.1	n.s.	< 0.01	< 0.0001	n.s.
UPDRS IV	3.5 ± 2.0	2.9 ± 2.8	6.3 ± 3.9	4.6 ± 2.6	n.s.	n.s.	< 0.01	< 0.05
Total UPDRS	51.4 ± 24.9	36.3 ± 13.2	70.3 ± 27.1	38.6 ± 14.2	< 0.05	< 0.01	< 0.0001	< 0.05
Smell	4.4 ± 3.6	4.6 ± 3.1	3.7 ± 1.9	5.7 ± 5.0	n.s.	n.s.	n.s.	n.s.
HY	2.5 ± 1.2	2.6 ± 0.7	3.0 ± 0.8	2.4 ± 0.6	n.s.	n.s.	< 0.05	n.s.
MMSE	27.6 ± 5.0	29.2 ± 1.4	25.0 ± 8.6	29.3 ± 1.2	n.s.	n.s.	n.s.	n.s.
MoCA-J	25.7 ± 3.0	26.1 ± 3.5	23.6 ± 4.1	26.2 ± 3.0	n.s.	n.s.	< 0.05	n.s.
FAB	15.7 ± 2.0	16.3 ± 1.5	15.3 ± 1.7	16.7 ± 1.7	n.s.	n.s.	n.s.	n.s.
LED (mg)	449 ± 174	390 ± 186	465 ± 206	497 ± 202	n.s.	n.s.	n.s.	n.s.
Stool frequency (/week)	2.6 ± 1.6	3.5 ± 1.3	3.0 ± 1.9	3.6 ± 1.8	n.s.	n.s.	n.s.	n.s.
LBP (ng/mL)	13225 ± 4073	12570 ± 3714	12620 ± 3540	12307 ± 3204	n.s.	n.s.	n.s.	n.s.

Mean ± standard deviation (SD) are indicated. *P*-value was calculated by Fisher’s exact test for genders and habitual consumption of fermented milk. Otherwise, *p*-value was calculated by Student’s unpaired *t*-test. Hyphen indicates that statistical significance is not applicalbe. BMI, Body Mass Index; UPDRS, Unified Parkinson’s Disease Rating Scale; Smell, score of the Odor Stick Identification Test for the Japanese (OSIT-J); HY, the Hoehn and Yahr scale; MMSE, Mini Mental State Examination; MoCA-J, the Japanese version of the Montreal Cognitive Assessment; FAB, the Frontal Assessment Battery at bedside; LED, Levodopa-equivalent dose; and LBP, lipopolysaccharide-binding protein.

### Clinical and laboratory features: Comparison between years 0 and 2

We next compared clinical features and serum LBP levels between years 0 and 2 in the deteriorated and stable groups (Table 1). The total UPDRS score, MOCA-J score, and HY score were significantly elevated only in the deteriorated group in 2 years. Serum LBP levels were not different between years 0 and 2 in either group, although LBP levels tended to decrease in the deteriorated group.

### Bacterial counts between the deteriorated and stable groups

**Fig 1:**
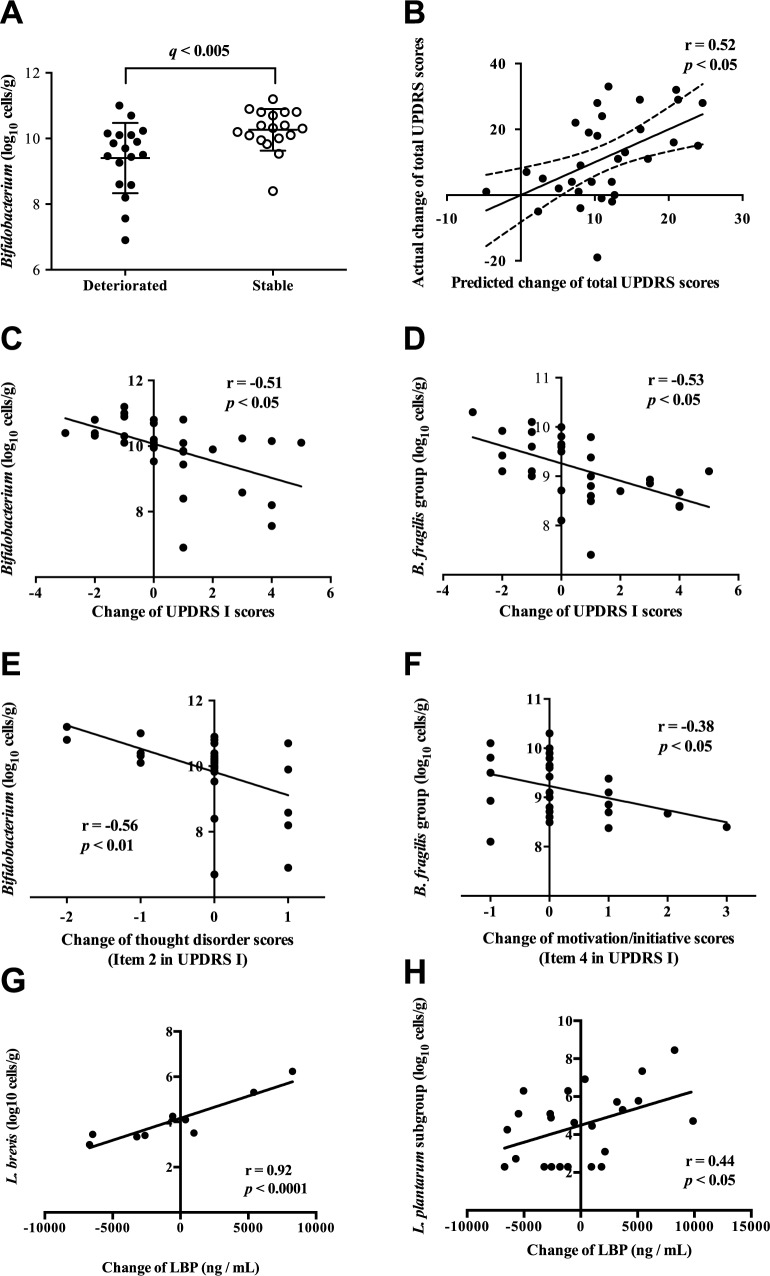
Prediction of worsening of UPDRS scores using gut microbiota at year 0. **(A)** Geometric plot of the counts of *Bifidobacterium* at year 0 in the deteriorated and stable groups. *P*-value was corrected by the Benjamini and Hochberg method to calculate the false discovery rate (FDR) (*q*-value). **(B)** A stepwise linear regression analysis for predicting a change of UPDRS scores in 2 years using the counts of 10 bacterial groups/genera/species at year 0 yielded the plotted prediction model, which is comprised of the counts of *Bifidobacterium* (standardized β = -0.45, *p* < 0.05) and *Atopobium* cluster (standardized β = 0.50, *p* < 0.01). **(C, D)** Correlation between a change of UPDRS I scores in 2 years and the count of *Bifidobacterium* at year 0 **(C)** and the count of *B*. *fragilis* group at year 0 **(D)**. **(E)** Correlation between a change of thought disorder scores (Item 2 in UPDRS I) in 2 years and the count of *Bifidobacterium* at year 0. **(F)** Correlation between a change of motivation/initiative scores (Item 4 in UPDRS I) in 2 years and the count of *B*. *fragilis* group at year 0. **(G, H)** Correlation between a change of LBP concentrations in 2 years and the count of *L*. *brevis* at year 0 **(G)** and the count of *L*. *plantarum* at year 0 **(H)**. **(B, C, D, E, and F)** Pearson’s correlation coefficients (r) are indicated with respective *p*-values.

The detection rate of each bacterium was not different between the deteriorated and stable groups at years 0 and 2. Similarly, there was no difference in total fecal bacterial counts between the 2 groups at years 0 and 2. The deteriorated group had lower *Bifidobacterium* counts (*q* < 0.05, FDR correction) than the stable group at year 0 (Fig 1A), suggesting that lower counts of *Bifidobacterium* at year 0 may be predictive of progression of PD in 2 years.

### Bacterial counts between years 0 and 2

Total fecal bacterial counts were decreased in 2 years in all PD patients, as well as in both the deteriorated and stable groups (Table 2 and S2 Table). In the deteriorated group, the count of *L*. *gasseri* subgroup became lower in 2 years (S2 Table). In the stable group, the counts of 7 bacteria (*Bifidobacterium*, *Clostridium leptum* subgroup, *Bacteroides fragilis* group, *Atopobium* cluster, *Enterococcus*, *L*. *gasseri* subgroup, and *Lactobacillus reuteri* subgroup) became lower in 2 years (Table 2 and S2 Table). When all PD patients were analyzed together, in addition to the 7 bacteria that were decreased in the stable group, *Prevotella* became lower in 2 years (Table 2 and S2 Table).

**Table 2:** Difference of bacterial counts between years 0 and 2.

	All PD patients (*n* = 28)			Deteriorated group (*n* = 11)			Stable group (*n* = 17)		
	Year 0	Year 2	*q*-value	Year 0	Year 2	*q*-value	Year 0	Year 2	*q*-value
Total fecal bacterial count	10.6 ± 0.3	10.0 ± 0.6	< 0.0001^c^	10.5 ± 0.3	10.2 ± 0.3	< 0.05^c^	10.7 ± 0.3	9.9 ± 0.7	< 0.0005^c^
*Bifidobacterium*	9.9 ± 1.0	9.0 ± 1.0	< 0.0001	9.4 ± 1.1	9.1 ± 1.1	n.s.	10.3 ± 0.6	8.9 ± 1.0	<0.001
*C*. *coccoides* group^a^	9.2 ± 0.5	9.0 ± 0.8	n.s.	9.2 ± 0.4	9.2 ± 0.6	n.s.	9.2 ± 0.6	8.9 ± 0.9	n.s.
*C*. *leptum* subgroup^a^	9.8 ± 0.8	9.2 ± 0.7	< 0.005	9.7 ± 1.0	9.4 ± 0.3	n.s.	9.9 ± 0.6	9.1 ± 0.8	< 0.005
*B*. *fragilis* group^a^	9.2 ± 0.7	8.6 ± 1.1	< 0.05	9.1 ± 0.5	8.8 ± 0.6	n.s.	9.3 ± 0.7	8.4 ± 1.3	< 0.01
*Atopobium* cluster	9.4 ± 0.5	9.0 ± 0.8	< 0.005	9.4 ± 0.6	9.3 ± 0.5	n.s.	9.3 ± 0.5	8.8 ± 0.9	< 0.01
*Prevotella*	6.7 ± 1.8	6.0 ± 1.2	< 0.05	7.0 ± 1.8	6.3 ± 1.4	n.s.	6.5 ± 1.7	5.8 ± 1.0	n.s.
Total *Lactobacillus*^*b*^	7.8 ± 1.2	7.8 ± 1.2	n.s.	8.0 ± 1.4	8.2 ± 1.1	n.s.	7.7 ± 1.3	7.5 ± 1.3	n.s.
Enterobacteriaceae	7.4 ± 0.8	7.0 ± 1.3	n.s.	7.5 ± 0.8	6.8 ± 1.5	n.s.	7.2 ± 0.8	7.1 ± 1.2	n.s.
*Enterococcus*	7.3 ± 1.1	6.1 ± 2.0	< 0.0001	7.0 ± 1.3	5.7 ± 2.0	n.s	7.3 ± 1.0	6.4 ± 2.0	< 0.05
*Staphylococcus*	4.6 ± 1.0	4.6 ± 1.1	n.s.	4.6 ± 1.1	4.5 ± 1.1	n.s.	4.5 ± 1.1	4.7 ± 1.1	n.s.

Mean and SD are indicated. *P*-value was first calculated by Wilcoxon signed-rank test, and was corrected by the Benjamini and Hochberg method to calculate the false discovery rate (FDR) (*q*-value).

^a^The counts of the three bacterial groups/subgroups were significantly lower at year 0 in PD patients compared to controls [18].

^b^The counts of total *Lactobacillus* was significantly higher at year 0 in PD patients compared to controls [18].

^c^For comparison of total fecal bacterial counts, *p*-value not *q*-value is indicated.

### Correlations between the bacterial counts at year 0 and the progression of clinical symptoms in 2 years

We predicted a change of total UPDRS scores in 2 years using fecal bacterial counts at year 0 using multiple linear regression models with stepwise selection. Among 19 bacterial groups/genera/species in YIF-SCAN, *Clostridium perfringens* and *Pseudomonas* were excluded from the analysis because of low detection rates in both groups. In addition, the counts of 8 groups/subgroups in *Lactobacillus* were combined together to calculate the count of total *Lactobacillus*. We thus used 10 representative bacterial groups/genera/species to make regression models. The counts of *Bifidobacterium* (standardized β = -0.45, *p* < 0.05) and *Atopobium* cluster (standardized β = 0.50, *p* < 0.01) at year 0 predicted a change of total UPDRS scores with r = 0.52 (*p* < 0.05) (Fig 1B). Among UPDRS I, II, III, and IV, only changes of UPDRS I scores were negatively correlated with the count of *Bifidobacterium* (r = -0.51, *p* < 0.05) (Fig 1C) and *B*. *fragilis* group (r = -0.53, *p* < 0.05) (Fig 1D). Further dissection of UPDRS I into each item revealed that worsening of thought disorder (Item 2 in UPDRS I), representing hallucinations and delusions, had a negative correlation with the count of *Bifidobacterium* (r = -0.56, *p* < 0.01) (Fig 1E). Similarly, worsening of motivation/initiative scores (Item 4 in UPDRS I) had a negative correlation with the count of *B*. *fragilis* group (r = -0.38, *p* < 0.05) (Fig 1F).

### Correlations between the bacterial counts at year 0 and the change of serum LBP levels in 2 years

In order to examine whether bacterial counts at year 0 are able to predict up- or down-regulation of serum LBP levels in 2 years, we analyzed correlations between the two factors. The changes of serum LBP were positively correlated with the counts of *L*. *brevis* (r = 0.92) and *L*. *plantarum* subgroup (r = 0.44) at year 0 (Fig 1G and 1H).

## Discussion

Emergence of intestinal α-synuclein in aged non-PD subjects [32], as well as in presymptomatic PD patients [33], implies that the PD pathology starts from the intestine. Accumulating evidence suggests involvement of intestinal microbiota in the development of intestinal α-synuclein [9–11]. We [18] and others [12–17, 19, 20] indeed reported intestinal dysbiosis in PD patients. However, the effects of intestinal microbiota on progression of PD have never been addressed. In an effort to disclose intestinal bacteria that are associated with progression of PD, we examined the effects of intestinal microbiota at year 0 on the progression of PD evaluated at years 0 and 2.

We evenly divided the 36 patients into the deteriorated and stable groups based on a change in the total UPDRS scores in 2 years. Higher total UPDRS scores and higher LEDs even at year 0 in the deteriorated group suggest that the deteriorated group already had advanced PD pathology at year 0. As the disease duration was rather shorter in the deteriorated group, the deteriorated group might have had accelerated PD pathology from the beginning. In addition, the difference in intestinal bacteria between the deteriorated and stable groups may be accounted for by the difference in genders in both groups. Although the deteriorated and stable groups were comprised of 44.4% and 77.8% females, respectively (Table 1), there was no statistical significance (*p* = 0.086 by Fisher's exact test) and bacterial counts at year 0 were not different between males and females [18].

In 2 years, total fecal bacterial count, as well as the counts of 6 out of 10 representative intestinal bacterial groups/genera/species, were decreased in all PD patients (Table 2). A decreased number of total fecal bacteria was previously reported in patients with constipation-type irritable bowel syndrome [34] and aged subjects [35]. The number of bacteria decreased in 2 years was more in the stable group (4 out of 10 bacteria) compared to the deteriorated group (0 out of 10 bacteria). As far as we know, no other reports have addressed temporal profiles of gut microbiota in PD patients. We previously reported that the counts of *C*. *coccoides* group, *C*. *leptum* subgroup, and *B*. *fragilis* group were lower in PD patients than healthy cohabitants [18]. The counts of *Bacteroides* were also low in PD in Germany [12], China [20] and Russia [19], but not in Finland [17] or the USA [15], and high in the USA [14]. In contrast, the counts of *Clostridium* were rather high in PD in Finland [17], low in Germany [13] and the USA [14, 15], and not different in Northern Germany [14]. There seems no intestinal bacterium that is specifically changed in PD. Among the 3 bacteria that were low in PD in our previous study [18], the counts of *C*. *leptum* subgroup and *B*. *fragilis* group were further decreased in 2 years in the stable group, but not in the deteriorated group. One possibility is that there are lower plateau levels for these 2 bacteria, and that the deteriorated group might have already come close to the plateau levels at year 0. Alternative possibility is that the counts of *C*. *leptum* subgroup and *B*. *fragilis* group were indeed decreased in both groups, but statistical significance was observed only in the stable group by chance. Similar to *C*. *leptum* subgroup and *B*. *fragilis* group, which were decreased in the stable group, the count of *C*. *coccoides* group was also decreased in the stable group but without statistical significance. Thus, the count of *C*. *coccoides* group may also rapidly decrease early in the course of PD. In contrast to the 3 bacteria, the counts of total *Lactobacillus* were higher in PD patients compared to healthy cohabitants in our previous study [18]. *Lactobacillus*, however, remained unchanged in 2 years in either group. Reevaluation of the count of *Lactobacillus* at year 0 and the degree of constipation at year 0 revealed that the correlation coefficient between *Lactobacillus* and stool frequency was -0.475 (*p* < 0.001), indicating that *Lactobacillus* was increased with constipation. Lack of change in the severity of constipation in 2 years in both groups (Table 1) may be correlated with lack of change in the counts of *Lactobacillus*.

Retrospective analysis of intestinal microbiota at year 0 revealed that the deteriorated group had a lower count of *Bifidobacterium* compared to the stable group (Fig 1A), although we previously reported no difference between PD patients and healthy cohabitants at year 0 [18]. Similar to *C*. *leptum* subgroup and *B*. *fragilis* group stated above, the *Bifidobacterium* counts at year 2 were not different between the deteriorated and stable groups (Table 2). This may suggest that the *Bifidobacterium* count rapidly decreases in patients with accelerated PD pathology. Presumptive differential declines of the counts of *C*. *leptum* subgroup, *B*. *fragilis* group, and *Bifidobacterium* between the deteriorated and stable groups suggest that gut microbiota may be critically influenced by the time after onset. Indeed, we previously reported that the count of *C*. *coccoides* was lower in PD patients compared to healthy cohabitants [18]. However, the counts of *C*. *coccoides* were not different between the deteriorated and stable groups at years 0 and 2 in our current study. The count of *C*. *coccoides* might have been decreased shortly after the onset of PD in the deteriorated group, whereas the count might have been decreased sometime after the onset of PD in the stable group. The time when we analyzed the counts of *C*. *coccoides* in the two groups might have been too late to detect the difference.

Stepwise linear regression analysis enabled us to make a model to predict a change of total UPDRS scores in 2 years using *Bifidobacterium* and *Atopobium* at year 0 (Fig 1B). However, no individual bacterial groups/genera/species at year 0 were correlated with worsening of total UPDRS scores in 2 years. Nonetheless, a low count of *Bifidobacterium* at year 0 was associated with worsening of UPDRS I (Fig 1C), especially worsening of hallucination/delirium (Fig 1E), in 2 years. Similarly, a low count of *B*. *fragilis* at year 0 was associated with worsening of UPDRS I (Fig 1D), especially lack of motivation/initiative (Fig 1F), in 2 years. While *Bifidobacterium* constitutes the largest group in the infant intestine, *Bifidobacterium* becomes the third or fourth largest group in the adult intestine and gradually declines with aging [36]. We indeed showed that *Bifidobacterium* constituted the fourth largest group in healthy cohabitants [18]. Formula-fed babies have a lower number of *Bifidobacterium* than breast-fed babies [37], which might account for a higher risk of diarrhea and allergies in formula-fed infants [38]. Probiotic administration of *Bifidobacterium* species ameliorates irritable bowel syndrome [39]. *Bifidobacterium* produces lactic acid, which regulates intestinal microbial homeostasis, inhibits growth of pathogenic and harmful bacteria, and modulates local and systemic immune responses [40]. *Bifidobacterium* additionally produces vitamins B1, B2, and K [41]. Probiotic administration of *Bifidobacterium* for 12 weeks has favorable effects on MMSE score, malondialdehyde, hs-CRP, markers of insulin metabolism, and triglycerides levels in patients with Alzheimer’s disease (AD) [42]. We previously reported that an oxidative stress marker (urine 8-OHdG) is elevated in PD patients with hallucination [43]. As UPDRS I represents severities of mentation, behavior, and mood including hallucination, the beneficial effects of *Bifidobacterium* on PD and AD, if any, may be partly attributed to its anti-oxidant effect. *Bacteroides* also constitutes one of the most dominant bacterial species in mammalian gastrointestinal tract. Transplantation of human *Bacteroides* to the mouse intestine increases the degradation efficiency of branched chain amino acids (BCAA) in the gut, reduces production of BCAA, and decreases circulating levels of BCAA [44]. Since BCAAs compete for entering into the brain with aromatic amino acids (phenylalanine and tyrosine), the level of aromatic acids in the brain decreases, which is followed by a decreased serotonin (5-HT) level in the brain. Worsening of UPDRS I in patients with low *Bacteroides* counts may represent decreased serotonin levels in the brain.

Low concentrations of LBP enhance LPS-induced expression of interleukin (IL)-1 and tumor necrosis factor (TNF)-α in membrane-bound CD14 (mCD14)-positive cells, or activates expression of IL-6 and IL-8 in mCD14-negative cells. In contrast, high concentrations of LBP neutralize bacterial endotoxin, and downregulates expression of proinflammatory cytokines such as TNF-α [45]. *L*. *plantarum* subgroup has lipoteichoic acid, which constitutes a major cell wall component of this species. Lipoteichoic acid of *L*. *plantarum* exerts anti-inflammatory effects on human intestinal epithelial cells by blocking IL-8 production [46]. Similarly, *L*. *brevis* improves intestinal barrier function in in a murine model of colitis [47]. Oral administration of *L*. *brevis* in aged mice for 8 weeks reduces the LPS level in the colon fluid and the blood, and inhibits the expression of inflammatory markers such as myeloperoxidase, TNF-α, and IL-1β, and suppresses NF-κB activation [48]. Elevation of serum LBP in 2 years in PD patients with high intestinal counts of *L*. *brevis* and *L*. *plantarum* subgroup suggests that these bacteria are likely to be protective against production of proinflammatory cytokines. We previously analyzed the serum levels of IL-6 and TNF-α [18] As we observed no difference in these cytokines between PD and control groups, we did not measure them at year 2. Our current study additionally revealed that there was no difference in these cytokines at year 0 between the deteriorated and stable groups.

As speculated in all sporadic diseases, PD is likely to be caused by an interaction between genetic and environmental factors. According to meta-analysis, prevalence of PD in 70- to 79-year-old population in Asia is significantly lower than those in North America, Europe, and Australia [49]. Similarly, prevalence of PD in all other age groups in Asia is low compared to other regions. *Bifidobacterium* is dominant in Asian people compared to Western people [50]. We showed that a low count of *Bifidobacterium* was associated with worsening of PD symptoms. *Bifidobacterium* may be protective against progression of PD. However, *Bifidobacterium* was not different between PD patients and controls in Japan [18] and the USA [15]. Additionally, *Bifidobacterium* was rather higher in PD patients than controls in Germany [12]. A small number of PD patients precluded us from drawing a decisive conclusion. Studies with a larger cohort for a longer time in multiple countries using the identical method for intestinal microbiota analysis [51] may elucidate the causal association of *Bifidobacterium* and other bacteria in the development and progression of PD.

## Supporting information

S1 Table Primer sequences used in this study. (DOCX)

S2 Table Change of the counts of *Lactobacillus* subgroup/species, as well as bacteria with low detection rates, between years 0 and 2. (DOCX)
