# Supplementary material for: Progression of Parkinson's disease is associated with gut dysbiosis: Two-year follow-up study
Source: PLoS One. 2017 Nov 1;12(11):e0187307. doi: 10.1371/journal.pone.0187307 (PMC5665539; doi:10.1371/journal.pone.0187307)
Supplement: S1 Table — (DOCX) [file pone.0187307.s001.docx]

**Supplementary Table 1. Primer sequences used in this study**

| Target bacteria^a^ | Primer | Sequence (5’ - 3’) | Reference |
| --- | --- | --- | --- |
| *Clostridium coccoides* group | g-Ccoc-F | AAATGACGGTACCTGACTAA | 4 |
|  | g-Ccoc-R | CTTTGAGTTTCATTCTTGCGAA |  |
| *Clostridium leptum* subgroup | sg-Clept-F | GCACAAGCAGTGGAGT | 5 |
|  | sg-Clept-R3 | CTTCCTCCGTTTTGTCAA |  |
| *Bacteroides fragilis* group | g-Bfra-F2 | AYAGCCTTTCGAAAGRAAGAT | 6 |
|  | g-Bfra-R | CCAGTATCAACTGCAATTTTA | 4 |
| *Bifidobacterium* | g-Bifid-F | CTCCTGGAAACGGGTGG | 4 |
|  | g-Bifid-R | GGTGTTCTTCCCGATATCTACA |  |
| *Atopobium* cluster | g-Atopo-F | GGGTTGAGAGACCGACC | 5 |
|  | g-Atopo-R | CGGRGCTTCTTCTGCAGG |  |
| *Prevotella* | g-Prevo-F | CACRGTAAACGATGGATGCC | 4 |
|  | g-Prevo-R | GGTCGGGTTGCAGACC |  |
| *Clostridium perfringens* | s-Clper-F | GGGGGTTTCAACACCTCC | 2 |
|  | ClPER-R | GCAAGGGATGTCAAGTGT | 3 |
| *Lactobacillus gasseri* subgroup | sg-Lgas-F | GATGCATAGCCGAGTTGAGAGACTGAT | 2 |
|  | sg-Lgas-R | TAAAGGCCAGTTACTACCTCTATCC |  |
| *Lactobacillus brevis* | s-Lbre-F | ATTTTGTTTGAAAGGTGGCTTCGG | 2 |
|  | s-Lbre-R | ACCCTTGAACAGTTACTCTCAAAGG |  |
| *Lactobacillus casei* subgroup | sg-Lcas-F | ACCGCATGGTTCTTGGC | 2 |
|  | sg-Lcas-R | CCGACAACAGTTACTCTGCC |  |
| *Lactobacillus fermentum* | LFer-1 | CCTGATTGATTTTGGTCGCCAAC | 2 |
|  | LFer-2 | ACGTATGAACAGTTACTCTCATACGT |  |
| *Lactobacillus plantarum* subgroup | sg-Lpla-F | CTCTGGTATTGATTGGTGCTTGCAT | 2 |
|  | sg-Lpla-R | GTTCGCCACTCACTCAAATGTAAA |  |
| *Lactobacillus reuteri* subgroup | sg-Lreu-F | GAACGCAYTGGCCCAA | 2 |
|  | sg-Lreu-R | TCCATTGTGGCCGATCAGT |  |
| *Lactobacillus ruminis* subgroup | sg-Lrum-F | CACCGAATGCTTGCAYTCACC | 2 |
|  | sg-Lrum-R | GCCGCGGGTCCATCCAAAA |  |
| *Lactobacillus sakei* subgroup | sg-Lsak-F | CATAAAACCTAMCACCGCATGG | 2 |
|  | sg-Lsak-R | TCAGTTACTATCAGATACRTTCTTCTC |  |
| Enterobacteriaceae | En-lsu-3F | TGCCGTAACTTCGGGAGAAGGCA | 1 |
|  | En-lsu-3'R | TCAAGGACCAGTGTTCAGTGTC |  |
| *Enterococcus* | g-Encoc-F | ATCAGAGGGGGATAACACTT | 2 |
|  | g-Encoc-R | ACTCTCATCCTTGTTCTTCTC |  |
| *Staphylococcus* | g-Staph-F | TTTGGGCTACACACGTGCTACAATGGACAA | 2 |
|  | g-Staph-R | AACAACTTTATGGGATTTGCWTGA |  |
| *Pseudomonas* | PSD7F | CAAAACTACTGAGCTAGAGTACG | 1 |
|  | PSD7R | TAAGATCTCAAGGATCCCAACGGCT |  |

^a^Group-, genus-, and species-specific primer sets amplify 16S rDNA sequences, except for En-lsu-3F/3’R, which amplifies 23S rDNA.

**References**

1. Matsuda K, Tsuji H, Asahara T, Kado Y, Nomoto K. Sensitive quantitative detection of commensal bacteria by rRNA-targeted reverse transcription-PCR. Appl Environ Microbiol. 2007;73(1):32-9. doi: 10.1128/AEM.01224-06. PubMed PMID: 17071791; PubMed Central PMCID: PMC1797142.
2. Matsuda K, Tsuji H, Asahara T, Matsumoto K, Takada T, Nomoto K. Establishment of an analytical system for the human fecal microbiota, based on reverse transcription-quantitative PCR targeting of multicopy rRNA molecules. Appl Environ Microbiol. 2009;75(7):1961-9. doi: 10.1128/AEM.01843-08. PubMed PMID: 19201979; PubMed Central PMCID: PMC2663197.
3. Kikuchi E, Miyamoto Y, Narushima S, Itoh K. Design of species-specific primers to identify 13 species of Clostridium harbored in human intestinal tracts. Microbiol Immunol. 2002;46(5):353-8. Epub 2002/07/26. PubMed PMID: 12139395.
4. Matsuki T. [Development of quantitative PCR detection method with 16S rRNA gene-targeted genus- and species-specific primers for the analysis of human intestinal microflora and its application]. Nihon Saikingaku Zasshi. 2007;62(2):255-61. Epub 2007/06/20. PubMed PMID: 17575792.
5. Matsuki T, Watanabe K, Fujimoto J, Kado Y, Takada T, Matsumoto K, et al. Quantitative PCR with 16S rRNA-gene-targeted species-specific primers for analysis of human intestinal bifidobacteria. Appl Environ Microbiol. 2004;70(1):167-73. Epub 2004/01/09. PubMed PMID: 14711639; PubMed Central PMCID: PMCPMC321263.
6. Matsuki T, Watanabe K, Fujimoto J, Miyamoto Y, Takada T, Matsumoto K, et al. Development of 16S rRNA-gene-targeted group-specific primers for the detection and identification of predominant bacteria in human feces. Appl Environ Microbiol. 2002;68(11):5445-51. Epub 2002/10/31. PubMed PMID: 12406736; PubMed Central PMCID: PMCPMC129894.
