# Supplementary material for: Progression of Parkinson's disease is associated with gut dysbiosis: Two-year follow-up study
Source: PLoS One. 2017 Nov 1;12(11):e0187307. doi: 10.1371/journal.pone.0187307 (PMC5665539; doi:10.1371/journal.pone.0187307)
Supplement: S2 Table — (DOCX) [file pone.0187307.s002.docx]

**Supplementary Table 2. Change of the counts of *Lactobacillus* subgroup/species, as well as bacteria with low detection rates, between years 0 and 2**

|  | All PD patients | | |  | Deteriorated group (*n* = 11) | | |  | Stable group (*n* = 17) | | |
| --- | --- | --- | --- | --- | --- | --- | --- | --- | --- | --- | --- |
|  | Year 0 | Year 2 | *q*-value |  | Year 0 | Year 2 | *q*-value |  | Year 0 | Year 2 | *q*-value |
| *L. gasseri* subgroup | 7.1 ± 1.3 | 6.2 ± 1.4 | < 0.0001 |  | 6.9 ± 1.6 | 6.3 ± 1.4 | < 0.01 |  | 7.1 ± 1.4 | 6.2 ± 1.4 | < 0.05 |
| *L. brevis* | 3.1 ± 1.3 | 3.6 ± 1.5 | n.s |  | 3.1 ± 1.1 | 3.0 ± 1.0 | n.s. |  | 3.2 ± 1.4 | 3.5 ± 1.7 | n.s. |
| *L. casei* subgroup | 5.5 ± 1.4 | 5.8 ± 1.4 | n.s |  | 5.1 ± 1.7 | 5.8 ± 1.2 | n.s. |  | 5.6 ± 1.4 | 5.8 ± 1.6 | n.s. |
| *L. fermentum* | 6.1 ± 1.5 | 6.1 ± 1.8 | n.s |  | 5.9 ± 1.7 | 6.1 ± 2.0 | n.s. |  | 5.9 ± 1.6 | 6.1 ± 1.8 | n.s. |
| *L. plantarum* subgroup | 4.5 ± 1.9 | 4.2 ± 1.6 | n.s |  | 4.6 ± 1.6 | 4.3 ± 1.4 | n.s. |  | 4.3 ± 2.0 | 4.2 ± 1.7 | n.s. |
| *L. reuteri* subgroup | 7.0 ± 1.7 | 5.8 ± 1.8 | < 0.0005 |  | 6.9 ± 2.0 | 6.4 ± 1.5 | n.s. |  | 6.9 ± 1.7 | 5.4 ± 1.9 | < 0.05 |
| *L. ruminis* subgroup | 5.9 ± 2.6 | 5.0 ± 2.7 | n.s |  | 6.2 ± 2.7 | 5.7 ± 3.0 | n.s. |  | 5.2 ± 2.5 | 4.6 ± 2.5 | n.s. |
| *L. sakei* subgroup | 4.2 ± 1.7 | 3.6 ± 1.8 | n.s |  | 3.9 ± 1.6 | 3.0 ± 1.2 | n.s. |  | 4.5 ± 1.9 | 3.9 ± 2.0 | n.s. |
| *C. perfringens* | 3.4 ± 1.4 | 3.5 ± 1.3 | n.s |  | 3.8 ± 1.7 | 3.7 ± 1.6 | n.s. |  | 3.2 ± 1.0 | 3.3 ± 1.1 | n.s. |
| *Pseudomonas* | 3.6 ± 0.7 | 3.6 ± 0.7 | n.s |  | 3.7 ± 0.9 | 3.6 ± 0.6 | n.s. |  | 4.5 ± 1.1 | 4.5 ± 1.2 | n.s. |

Values are shown in log_10_ cells/g of stool. Mean and SD are indicated. *P*-value was calculated by Wilcoxon signed-rank test, and was corrected by the Benjamini and Hochberg method to calculate the false discovery rate (FDR) (*q*-value).
